# The role of Themis in development of type 2 diabetes

**DOI:** 10.21203/rs.3.rs-7943370/v1

**Published:** 2025-11-07

**Authors:** Nicholas Gascoigne, Lukasz Wojciech, Mukul Prasad, Joanna Brzostek, Vasily Rybakin, John Hoerter, Bowen Hou, Desmond Tung, Yen Leong Chua, Jeanette Ampudia, Anooja Rai, Grzegorz Chodaczek, Guo Fu, Sven Pettersson

**Affiliations:** Immunology Center of Georgia, Augusta University; Łukasiewicz – PORT,; National University of Singapore; National University of Singapore; National University of Singapore; Novartis Biomedical Research; Augusta University; National University of Singapore; National University of Singapore; The Scripps Research Institute; National University of Singapore; Łukasiewicz – PORT,; Xiamen University; Karolinska Institutet

## Abstract

Type 2 diabetes (T2D) is a complex metabolic disorder driven by chronic inflammation and immune dysregulation, particularly within adipose tissue. This study investigates the role of the T cell-specific protein Themis in modulating immune-metabolic interactions that contribute to T2D pathogenesis. Using high-fat diet (HFD)-induced obesity models, we demonstrate that *Themis*-deficient (KO) mice exhibit accelerated weight gain, glucose intolerance, and insulin resistance compared to wild-type (WT) controls. These metabolic abnormalities are linked to functional alterations in the CD8^+^ T cell compartment, including site-specific clonal expansion and reshaping of the T cell receptor (TCR) repertoire within adipose tissue, suggesting antigen-driven activation. Additionally, *Themis* deficiency leads to significant shifts in gut microbiome composition, characterized by reduced diversity and increased abundance of *Firmicutes*, particularly *Clostridium* species. However, fecal microbiota transplantation from *Themis* KO mice into germ-free WT hosts failed to recapitulate the full T2D phenotype, underscoring the dominant role of intrinsic immune dysfunction over microbial dysbiosis. These findings highlight a synergistic interplay between adaptive immunity and the microbiome in shaping metabolic outcomes and suggest that T cells play a central role in responses that influence T2D progression. Our data advocate for a more integrated approach to T2D research, incorporating genetic, immunological, and microbial factors.

## Introduction

Type 2 diabetes (T2D) is a multifactorial metabolic disorder characterized by insulin resistance and chronic low-grade inflammation, often linked to obesity and immune dysregulation. Infiltration of adipose tissue by macrophages, which together with adipocytes form a major source of proinflammatory cytokines, is believed to be a key mechanism contributing to the development of insulin resistance^1–4^. These macrophages undergo a phenotypic shift to a proinflammatory M1-like state in obese individuals, amplifying local inflammation. The resulting cytokine milieu, including TNF, IL6, and MCP1, interferes with insulin signaling pathways in adipose tissue, liver, and muscle. Beyond innate immunity, the adaptive immune system plays a pivotal role in sustaining this proinflammatory state. Notably, the regulatory T cell (Treg) compartment is compromised both at the population level, reflected in an altered Treg to conventional T cell (Tconv) ratio, and at the clonal level, where individual Treg cells exhibit intrinsic functional impairments^4–9^. These defects further exacerbate immune imbalance and contribute to the pathogenesis of T2D. Another subset of T cells implicated in the development of insulin resistance is the cytotoxic CD8^+^ T cell population. In obese adipose tissue, activated CD8^+^ T cells engage with components of the innate immune system, promoting the polarization of macrophages toward a proinflammatory M1-like phenotype^10^. This interaction amplifies local inflammation and contributes to the disruption of insulin signaling. Moreover, CD8^+^ T cells that produce IFNγ have been linked to the regulation of metabolic homeostasis. Their activity influences the functional landscape of white adipose tissue (WAT), which comprises both classical white adipocytes and metabolically active beige adipocytes^11,12^. The latter share characteristics with brown adipocytes, particularly their response to cold exposure and their capacity to enhance fatty acid oxidation when activated^12,13^. This beige adipocyte function represents a critical axis in energy expenditure and metabolic regulation, which may be disrupted in the inflammatory milieu driven by IFNγ producing CD8^+^ T cells^14^.

Gut-resident microbial consortia play a pivotal role in regulating the host immune system. It is well established that members of these communities interact with various immune compartments, modulating responses to self and non-self-antigens^15–18^. Through these interactions, they can drive either pro-inflammatory or anti-inflammatory shifts across both the innate and adaptive arms of the immune system^15,16^. In both animal models and T2D patients, the gut microbiome undergoes notable compositional and functional shifts^19–22^. However, it is not fully understood how these changes affect the microbiome-immune system axis and how they contribute to the multifactorial landscape that defines the T2D phenotype.

While the role of the adaptive immune system in T2D pathogenesis has gained increasing attention, the molecular mechanisms underlying immune-metabolic crosstalk remain incompletely understood. The process of positive and negative selection of T cell receptors (TCRs) towards self-antigens takes place in the thymus. Themis is a T cell specific protein, which has been shown to regulate positive selection, and to set the TCR signaling threshold at this stage^23–31^. As a part of the TCR signalosome, Themis plays a pivotal role in interpreting TCR-pMHC interactions during thymic development and in peripheral responses to antigens^23,28–31^. In peripheral T cells, Themis integrates TCR signaling and cytokine responses^31^. Additionally, it has been shown that *Themis* knockout mice have defects in the TCR stimulation-induced up-regulation of insulin receptor, Glut1 and Glut6^32^. Beyond its critical function in translating TCR signals and setting the activation threshold of T cells, *Themis* SNPs have also been identified as associated with early-onset development of type 1 diabetes (T1D)^33–35^.

In this study, we investigate the impact of *Themis* deficiency on the development of insulin resistance and metabolic syndrome using high-fat diet (HFD)-induced obesity models in mice. Our findings reveal that *Themis* knockout (KO) mice exhibit accelerated weight gain, glucose intolerance, and insulin resistance compared to *Themis*-sufficient (wild-type: WT) controls. We have shown that these metabolic abnormalities are associated with functional alterations within the CD8^+^ T cell compartment. Through TCR repertoire analysis, we uncover enhanced site-specific clonal expansions of CD8^+^ T cells in *Themis*-deficient mice, suggesting antigen-driven activation within adipose tissue. Furthermore, we explore the interplay between *Themis*-dependent immune modulation and gut microbiome composition. *Themis* KO mice harbor a distinct microbial community with reduced diversity and altered taxonomic profiles in comparison to its WT counterpart. When transferred to *Themis*-sufficient, germ-free hosts, the *Themis* KO-derived microbiome fails to recapitulate the T2D phenotype observed in the *Themis*-deficient model. These results highlight the central role of CD8^+^ T cell activation in driving T2D pathogenesis, while also indicating that microbial dysbiosis contributes to disease progression, albeit not as a primary factor. These findings offer valuable insight into the complex interplay between the microbiome and immune system, deepening our understanding of their roles in the development of T2D. Also, our data emphasize the need for a more holistic approach in T2D research and diagnosis, one that considers genetic and immunological factors alongside microbial and metabolic parameters.

## Results

### Themis KO mice develop insulin resistance faster compared to Themis WT mice

**Figure 1:**
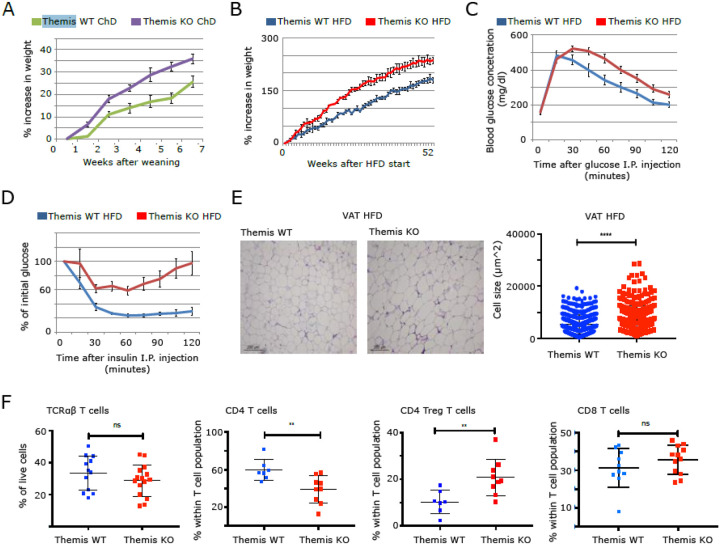
Development of metabolic disorder in *Themis* KO mice. **A**. Weight gain changes observed in *Themis* KO and *Themis* WT mice on normal chow diet (ChD). **B**. Weight gain changes observed in *Themis* KO and WT mice on high fat diet (HFD). **C**. Glucose tolerance at 12 weeks of HFD and **D** Insulin tolerance tests at 30 weeks of HFD to test glucose and insulin sensitivity of *Themis* KO and WT mice on high fat diet. **E**. (right) H&E-stained sections of VAT excised from *Themis* KO and WT mice on HFD. (left) Histogram summary of the adipocyte cell size in VAT of *Themis* KO and WT mice on high fat diet. 12 mice per genotype were used. Data representative of three independent experiments. **F**. Proportions of T cells, CD4^+^ T cells, Tregs and CD8^+^ T cells, in VAT of *Themis* KO and WT mice on HFD.

Several studies have identified a link between *THEMIS* polymorphisms and autoimmune diseases that may contribute to metabolic dysfunction^34–45^. We observed that *Themis* KO mice fed a normal chow diet exhibited increased weight gain compared to WT controls (Fig. 1A), suggesting a potential role for *Themis* in the regulation of body weight and the development of obesity-related metabolic disorders. We employed a high fat diet (HFD)-induced obesity model on both *Themis* WT and *Themis* KO genotypes, to investigate the whole body metabolism in a context of *Themis* deficiency. The diet intervention started at 6 weeks of age and from this moment mice from each cohort were weighed weekly. Glucose and insulin tolerance tests were conducted at the 52-week endpoint of the experiment to evaluate insulin sensitivity in mice fed the HFD. The results show that KO mice on HFD gained weight faster (Fig. 1B), were more glucose intolerant (Fig. 1C), and more insulin resistant compared to WT mice (Fig. 1D). Histological analysis of visceral adipose tissue (VAT) revealed a pronounced impact of *Themis* deficiency in HFD-fed animals. The KO mice exhibited marked adipocyte hypertrophy compared to WT controls. This can be interpreted as a hallmark of exacerbated chronic low-grade inflammation in the *Themis*-KO model. We hypothesize that by enhancing low-grade inflammation in VAT, *Themis* deficiency might potentially contribute to metabolic dysfunction and altered disease kinetics. Notably, liver histology remained unchanged between genotypes, suggesting a site-specific effect of *Themis* deficiency.

### T2D phenotype in Themis KO mice is not associated with T cell enrichment in VAT

**Figure 2:**
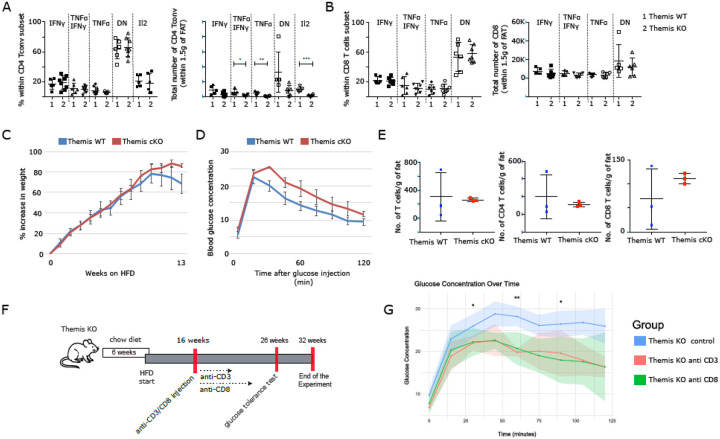
T cells as a potential driver of metabolic disorder in Themis KO model **A, and B**. Proinflammatory cytokine production in VAT of *Themis* KO and WT mice on HFD. **A and B** (left) Proportions and (right) number of CD4^+^ Tconvs and (E) CD8^+^ T cells from *Themis* KO and WT producing IFNg and TNF. The CD4^+^ Tconvs were additionally tested for IL2 production. DN refer to IFNg and TNF double negative status. Six WT and seven KO mice were examined and data has been pooled from three independent experiments. **C**. Weight gain changes and **D** Glucose tolerance test at 12 weeks of HFD to test glucose sensitivity of the dLck-Cre negative and positive (WT and conditional KO (cKO) respectively) mice on HFD. **E**. The total numbers of T cells, CD4^+^ T cells and CD8^+^ T cells per gram of fat in VAT of WT and cKO on HFD. Three littermate mice per genotype were used. Data were derived from a single experiment. **F** Timeline of diet intervention and antibody anti-CD3 and anti-CD8 treatment applied in T2D model with T cell depletion. **G**. Glucose tolerance test of anti-CD3 and anti-CD8 together with isotype treated control treated *Themis* KO mice on HFD.

Immune infiltration into the adipose tissue constitutes an early event that leads to inflammation and ultimately to T2D^5,6,46,47^. *Themis* is a T cell-specific protein, but its deletion in KO mice leads to pronounced insulin resistance compared to WT counterparts. This observation prompted us to hypothesize that disruptions in the adaptive immune system may contribute to the metabolic phenotype. Specifically, we considered that increased immune cell infiltration into VAT could underlie the observed dysfunction. To test this hypothesis, we analysed immune cell infiltration in VAT after 40 weeks of HFD. We isolated the stromal vascular fraction (SVF) and analysed it for the presence of pro-inflammatory T cells. Interestingly, we did not observe any clear difference in the proportion of total T cells, though there was a decrease in the proportion of total CD4^+^ T cells but not of CD8^+^ T cells in VAT of *Themis* KO mice (Fig. 1F). Additionally, we found an increase in the proportion of CD4^+^ Tregs in the VAT of *Themis* KO mice as compared to WT mice (Fig. 1F). However, upon quantifying total T cell numbers, including CD4^+^ Tconvs, Tregs, and CD8^+^ T cells, in *Themis* KO and WT mice, we observed a significant reduction of all subsets in the VAT of the knockout genotype. (Suppl. Figure 1A). Importantly, the *Themis* KO model is characterized by lymphopenia affecting both CD4^+^ and CD8^+^ T cells^23,28^. Therefore, the reduced number of total T cells observed in adipose tissue may simply reflect the systemic lymphopenic phenotype of *Themis*-deficient mice. Next, using PMA plus ionomycin activation, we looked at the potential of SVF T cells to produce cytokines. Notably, we did not find any statistically significant difference in the number of IFNg and TNF-producing T cells between KO and WT mice, except for a slight decrease in the number of Tconvs that were IFNg^+^, TNF^+^, IFNg^+^ TNF^+^ and IL2^+^ in VAT of *Themis* KO mice as compared to *Themis* WT mice (Fig. 2A, B). The reduction in CD4^+^ effector T cells was accompanied comparable number of CD8^+^ effector T cells in VAT between *Themis* KO and WT mice. Given the systemic lymphopenia associated with *Themis* deficiency, this suggests that CD8^+^ T cells may be selectively recruited, activated and polarized within adipose tissue in the absence of *Themis*. This observation raises the possibility that CD8^+^ T cells contribute to the development of the T2D phenotype in *Themis* KO mice. Finally, we checked the accumulation of macrophages in VAT and the polarization of these macrophages into M1-like or M2-like phenotypes. We didn’t observe any differences in either the accumulation of macrophages or their polarization in VAT of KO and WT mice (Suppl. Figure 1B).

### T2D phenotype in Themis KO mice is driven by CD8^+^ T cells

The lack of pronounced increase of effector T cell infiltration within adipose tissue was unexpected. To investigate whether *Themis*-dependent functional impairment of peripheral T cells contributes to the metabolic phenotype observed in *Themis* KO, we examined T2D development using a conditional *Themis* knockout model. Specifically, we utilized a previously described system in which mice carrying floxed *Themis* alleles (Themis^f/f^) were crossed with animals expressing the distal Lck-Cre transgene (dLck-Cre)^31^. In this model, dLck-Cre causes deletion of *Themis* only after positive and negative selection in the thymus^31^. Thus, any dysfunction will be restricted to peripheral T cells. Accordingly, the phenotype in this model upon HFD may help confirm or refute the role of peripheral T cell functionality as a key driver of metabolic dysregulation. The dLck-Cre negative and positive (referred to here as WT and conditional KO (cKO), respectively) mice cohorts were weighed weekly after starting the diet intervention at 6 weeks of age. The glucose tolerance test was done to assess insulin sensitivity of the HFD fed mice. Interestingly the trends regarding HFD-induced gained weight and glucose intolerance mirrored that observed in the germline *Themis* KO model. The cKO mice on HFD gained weight faster (Fig. 2C), and were more glucose intolerant (Fig. 2D), compared to the WT counterparts. We also looked at T cell infiltration in VAT of cKO and WT mice. When quantifying T cells per gram of adipose tissue, we again found no significant differences between cKO and WT mice (Fig. 2E). Despite the absence of overt effector T cell infiltration within the VAT of the cKO model, the collective data strongly support the hypothesis that *Themis* deficient T cells are key contributors to the development of the T2D phenotype. To more precisely investigate the contribution of specific T cell subsets to the development of the T2D phenotype, we performed *in vivo* antibody-mediated depletion in *Themis* KO. Animals received either anti-CD3 antibodies to deplete all T cells or anti-CD8 antibodies to selectively target CD8^+^ T cells (Fig. 2F). Mice were weighed at weekly intervals and glucose tolerance test was done to assess insulin sensitivity in mice treated with anti-CD3 and anti-CD8 antibodies. Notably, both anti-CD3 and anti-CD8 antibody injections markedly improved glucose clearance in KO mice compared to isotype controls (Fig. 2G). All these data strongly implicate CD8^+^ T cells as key drivers of the metabolic dysfunction observed in the *Themis*-deficient model.

### Unique shaping of the CD8^+^ T cell TCR repertoire upon homing to adipose tissue

**Figure 3:**
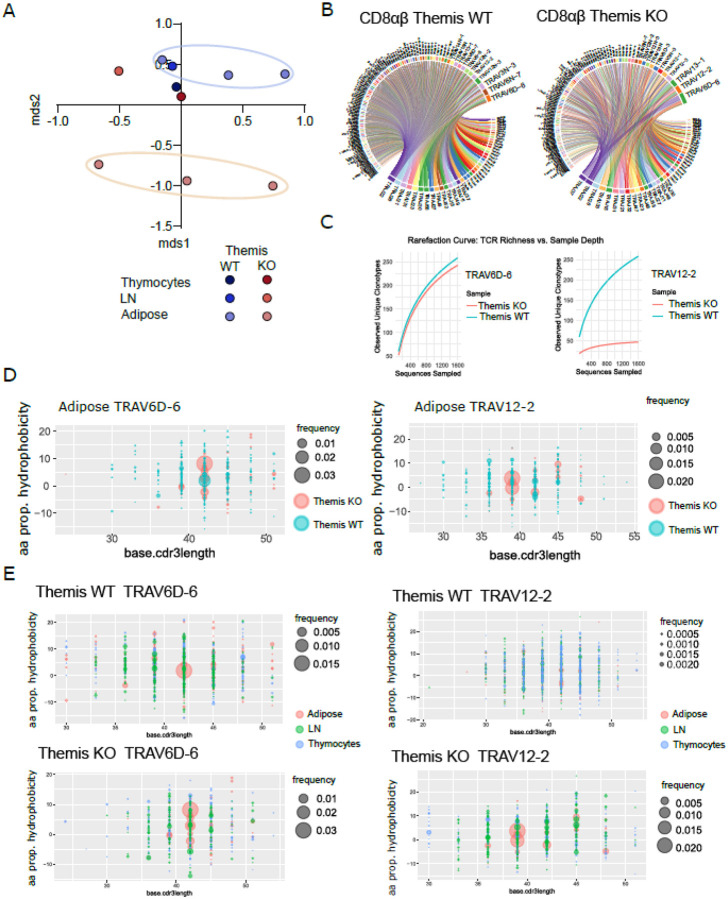
Site-specific expansion and physical properties of TCRa repertoires in WT and *Themis* KO **A**. Multidimensional scaling (MDS) plot illustrating the distribution of CD8αβ TCR repertoires in *Themis* WT and KO mice, highlighting TRAV families across samples. **B**. Overall comparison of TRAV and TRAJ usage, highlighting the presence of specific TRAV genes such as TRAV12–2, TRAV6D-6, and TRAV3N-3 across *Themis* WT and KO repertoires from adipose tissue. **C**. Rarefaction curves showing unique clonotype diversity across sampled sequences for *Themis*WT and KO, with TRAV12–2 exhibiting distinct site-specific expansion in *Themis*KO. **D**. Hydrophobicity and CDR3 length distribution of TRAV6D-6 and TRAV12–2 in adipose tissue, illustrating the frequency variation between genotypes. **E**. Physical properties (hydrophobicity and CDR3 length) of TRAV6D-6 and TRAV12–2 in adipose, thymocytes, and lymph nodes, comparing *Themis*WT and KO repertoires.

Our results indicate that T cells are the main factor orchestrating the kinetics of T2D development in the *Themis* KO model. Notably, both in humans and in mouse models of type T2D, the expansion of IFNγ-producing CD8^+^ T cells constitute a major component of the immune response^10,11^. This may contribute to adipose tissue inflammation and, consequently, to the progression of T2D. A key question is what mechanisms drive the expansion and differentiation of naïve CD8^+^ T cells into functional effectors within adipose tissue, and how *Themis* deficiency influences this process. Specifically, it remains unclear whether the acquisition of a proinflammatory phenotype by CD8^+^ T cells is driven by *in situ* recognition of specific antigens and subsequent polarization, or whether this process occurs independently of antigenic priming with adipose tissue-associated epitopes. To address this question, we analyzed the TCRα repertoires of CD8^+^ T cells isolated from two groups of animals (*Themis* WT and *Themis* KO), representing an advanced clinical stage of T2D induced by a HFD. The data sets included TCR repertoires from adipose tissue, lymph nodes (LN), and single-positive CD8^+^ thymocytes. When analyzed using multidimensional scaling (MDS), the repertoires derived from LN and thymocytes clustered together in both genotypes, indicating a degree of similarity between these T cell populations (Fig. 3A). Interestingly, the adipose tissue-derived repertoires were clearly separated from those of the LN and thymus, with this trend particularly pronounced in *Themis KO* animals (Fig. 3A). This provides clear evidence that during the homing of T cells into adipose tissue, these subsets undergo repertoire reshaping, likely driven by the unique antigenic environment present in this tissue. Furthermore, we hypothesize that *Themis* deficiency in CD8^+^ T cells, by altering the interpretation of TCR signaling, may lead to the activation and expansion of a broader range of clones in response to adipose tissue-associated antigens compared to *Themis* WT counterparts. This could help explain the differences in the kinetics of T2D development between *Themis* KO and WT models.

Next, we analyzed the usage of Vα and Jα segments within adipose tissue-derived TCR repertoires (Fig. 3B). We observed some similarities between the *Themis* WT and *Themis* KO groups. In both genotypes, the most dominant Vα segment was TRAV6D-6. However, TRAV6D-6 clones in *Themis* KO animals exhibited markedly lower diversity, as evidenced by their pairing with a significantly smaller number of Jα segments compared to TRAV6D-6 TCRs in the *Themis* WT group (Fig. 3B). Importantly, the repertoires of WT and KO animals exhibited substantial quantitative differences, as reflected by the distinct distribution of TRAV12–2 TCR clones. In *Themis* KO mice, this particular Vα segment was the second most abundant, whereas in *Themis* WT mice, it was detected at much lower frequency and did not rank among the dominant Vα segments (Fig. 3B).

The observation regarding the diversity of TRAV6D-6 TCRs in *Themis* WT and KO repertoires was further supported by a specific diversity analysis that incorporated information from the entire Vα chain, including the Vα and Jα segments as well as the CDR3 region. Indeed, the TRAV6D-6 component of TCR repertoire in *Themis* KO mice was less diverse than the repertoire in their *Themis* WT counterparts (Fig. 3B). The curves representing cumulative frequency distribution indicate enhanced clonal expansion of *Themis* KO T cells (Fig. 3C). This enhanced clonal expansion within adipose tissue was particularly strong in TRAV12–2 TCRs in *Themis* KO.

### Adipose tissue TCRs suggest in situ, antigen-driven CD8^+^ T cell expansion

The encounter between T cells expressing a particular TCR and antigen-presenting cells displaying their cognate antigen represents the initial stage of activation, eventually leading to clonal expansion. Importantly, the nature of the interaction (e.g. agonistic or antagonistic) between the TCR and the peptide-MHC complex is largely determined by the physical properties of the TCR’s CDR3 region. Thus, in the case of antigen-driven T cell proliferation, we would expect selective expansion of clones that exhibit specific physical traits in their CDR3 regions, such as lengths and amino acid compositions that influence hydrophobicity or charge distribution, which are optimally suited for recognizing and binding the presented antigen(s) with appropriate affinity and specificity. Given that our observations regarding TRAV6D-6 and TRAV12–2 TCRs may reflect patterns characteristic of clonal expansion, we analyzed the CDR3 regions from these two TCR groups, focusing on physical features that could provide insights into the nature of their antigen-driven selection within the adipose tissue milieu. In this analysis, we correlated CDR3 length with hydrophobicity, charge, and polarity, incorporating the frequency of each TCR clone within the repertoires to better understand the relationship between these biophysical parameters and clonal dominance. Interestingly, when comparing TRAV6D-6 TCRs from *Themis* WT and *Themis* KO groups, we found that in both repertoires, CDR3 regions of 14 amino acids in length (corresponding to 42 nucleotides in our graphs) were associated with expanded clones exhibiting a characteristic hydrophobicity index, polarity, and CDR3 charge (Fig. 3D). However, in the *Themis* KO group, this segment of the repertoire (TRAV6D-6 with 14-amino-acid CDR3s) displayed a markedly broader range of clones sharing specific physical properties. This suggests that a larger number of T cells in *Themis* KO mice responded to one or more antigens presented within the adipose tissue niche, compared to their WT counterparts. By applying the same strategy, we analysed TRAV12–2 TCRs from *Themis* WT and KO groups. For TRAV12–2 TCRs, a characteristic expansion of clones with particular physical features was only observed in *Themis* KO TCR repertoires and was restricted to CDR3 regions of 13 amino acids in length (corresponding to 39 nucleotides in our graphs) (Fig. 3D). Together, these findings suggest that the increased representation of the TRAV12–2 component within the *Themis* KO TCR repertoire depicted in Fig. 3B may result from the expansion of clones in response to one or more antigens present in the adipose tissue environment. Moreover, these data support our hypothesis that Themis deficiency leads to altered interpretation of TCR signaling, possibly resulting in a lowered activation threshold and, consequently, enhanced (quantitatively) clonal expansion of *Themis* KO T cells. This heightened activation and proliferation of T cells within adipose tissue may, in turn, contribute to the accelerated progression of T2D observed in *Themis* KO mice compared to their WT counterparts.

Given the clear trends observed in the CDR3 regions of TRAV6D-6 and TRAV12–2 TCRs, we next analyzed the distribution of physical features across TCR repertoires from *Themis* WT and KO groups. This time, we compared repertoires derived from thymocytes, lymph nodes (LN), and adipose tissue within each genotype individually. This strategy aimed to determine whether the expansion of clones with specific physical features was restricted to the adipose tissue or also present in other compartments. Notably, in both genotypes, expansion of TRAV6D-6 clones was observed exclusively in adipose tissue (Fig. 3E and Suppl. Figure 2A). A similar trend of site-specific expansion was seen for TRAV12–2 TCRs; however, in this case, such expansion was restricted to the adipose tissue of *Themis* KO animals, with no comparable tissue-specific enrichment in WT counterparts (Fig. 3E and Suppl. Figure 2B). Collectively, these data strongly support our hypothesis that the CD8^+^ T cell compartment may contribute to adipose tissue inflammation and, consequently, to the development of metabolic syndrome through recognition of adipose tissue-specific antigens. Additionally, in light of these findings, we propose that altered antigen recognition in the *Themis*-deficient model, along with a broader and more robust clonal response, largely accounts for the T2D phenotype observed in this genotype.

### Proinflammatory response of CD8 T cells in adipose tissue indicates classical MHC restriction

The site-specific expansion of TRAV6D-6 (in both genotypes) and TRAV12–2 (in *Themis* KO) suggests a predominantly classical MHC class Ia-restricted response. However, the possibility of a nonclassical MHC-class Ib-restricted mechanism contributing to T2D development in our mouse model cannot be excluded. Notably, TRAV9N-3 (encoding TCR Vα3.2), which recognizes insulin in the context of the MHC class Ib molecule Qa-1 (Qa-1b), has been successfully cloned and functionally tested, highlighting its potential role in T2D pathology^48,49^. Moreover, previous reports indicate a significant quantitative enrichment of TRAV9N-3 TCRs in the *Themis* KO model, as evidenced by the increased proportion of Vα3.2^+^ expressing cells within the CD8^+^ T cell repertoire^50^.

**Figure 4:**
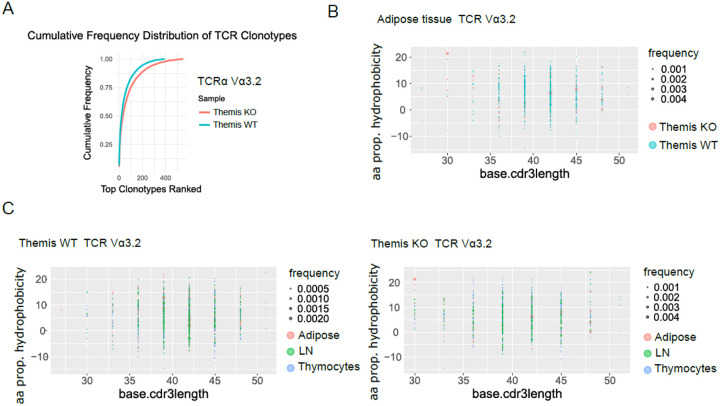
Functional and structural properties of TRAV9N-3 (Vα3.2) TCRs in *Themis*KO and WT mice **A**. Cumulative frequency distribution of TRAV9N-3/Vα3.2 clonotypes in *Themis* WT and KO mice, showing relative clonotype abundance across samples in VAT. **B**. Hydrophobicity index and CDR3 length distribution for TRAV9N-3/Vα3.2 TCRs within adipose tissue, comparing *Themis* KO and WT mice, with data points representing individual clonotypes. **C**. Comparison of hydrophobicity and CDR3 length within thymocytes, lymph nodes (LN), and adipose tissue across WT and KO genotypes, highlighting site-specific trends.

Interestingly, analysis of clonal diversity revealed that both *Themis* WT and KO repertoires from adipose tissue display comparable patterns. The cumulative frequency distribution of Vα3.2 TCR clonotypes (Fig. 4A), indicates that the process of repertoire reshaping upon homing into adipose tissue is similar across both genotypes. This suggests that the extent of qualitative and quantitative repertoire homing into adipose tissue, as well as potential clonal expansion within the adipose environment, is largely comparable between *Themis* WT and KO mice. To further explore this idea, we analyzed the CDR3 TRAV9N-3/Vα3.2 repertoires of *Themis* WT and KO using the same approach previously applied to TRAV6D-6 and TRAV12–2. Notably, the distribution of physical features within the CDR3 region did not reveal substantial clonal expansion, showing no restriction to a specific CDR3 length or distinct physical traits (Fig. 4B, Suppl. Figure 3A and B). This similarity in the physical mapping between genotypes suggests that *Themis* deficiency does not alter the range of antigen recognition by TRAV9N-3/Vα3.2 TCRs. To assess site-specific drift or potential enrichment of individual TCRs, we compared repertoires from both genotypes across thymocytes, lymph nodes, and adipose tissue. Notably, in both WT and KO TRAV9N-3/Vα3.2 TCR repertoires, we observed a similar distribution of CDR3 clones across all three organs, indicating a predominantly passive homing to each niche rather than antigen-driven repertoire reshaping (Fig. 4C, Suppl. Figure 3C and D). Furthermore, the lack of clonal expansion within adipose tissue suggests that TRAV9N-3/Vα3.2 repertoires do not contribute to the proinflammatory processes occurring in adipose tissue through antigen-restricted mechanisms. Finally, all these data collectively indicate that the proinflammatory shift of CD8^+^ T cells within adipose tissue is controlled by antigen recognition restricted to classical MHC class Ia.

### Themis KO mice exhibit a distinct gut microbiome compared to their WT counterparts

**Figure 5:**
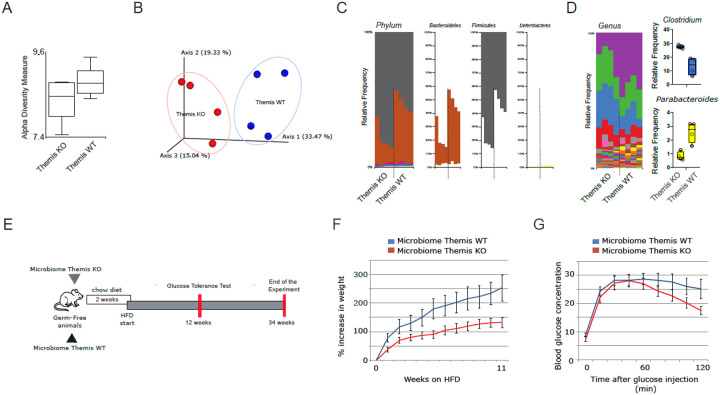
The microbiome established in *Themis*KO mice does not independently confer susceptibility to T2D **A**. Alpha diversity of the gut microbiomes isolated from *Themis* WT and KO mice. Estimation of the diversity indexes was performed on V3-V4 partial 16s sequencing data. **B**. Beta diversity analysis of gut microbiomes derived from *Themis* WT and KO groups. **C**. Relative abundance of dominant bacterial phyla in the gut microbiomes of *Themis*WT and KO mice. **D**. Relative abundance of key bacterial genera within the gut microbial communities of *Themis* KO and WT mice. **E**. The FMT timeline includes transplantation of biological material from *Themis* WT and KO mice into germ-free recipients, followed by dietary intervention within a T2D model. **F**. Weight gain changes in germ free mice conventionalized with microbiome from *Themis* WT and KOmice on HFD. **G**. Glucose Tolerance Test of germ-free mice conventionalized with microbiome from *Themis* WT and KO mice on HFD. Five germ free mice were used for FMT from either genotype.

The microbiome-host adaptive immune system interaction axis plays a critical role in maintaining physiological homeostasis. Therefore, the quantitative and qualitative dysfunctions of T cells associated with the *Themis* KO genotype are likely to alter the nature of host-microbiome interactions, which should be reflected in the taxonomic composition of microbial communities derived from *Themis* KO mice compared to their WT counterparts. To assess potential differences in the gut microbiota between the two mouse models, we collected stool samples from approximately eight-week-old males from each group. Using next-generation sequencing (NGS) of the V3-V4 regions of the 16S rRNA gene, we analyzed the taxonomic composition of bacterial communities derived from *Themis* WT and KO mice. Interestingly, despite the limited resolution of the data, restricted to the genus level, we were still able to observe genotype-associated differences in taxonomic distribution. The microbiome derived from the WT group exhibited higher alpha diversity than KO counterparts, indicating a more “pro-healthy” distribution of species (Fig. 5A). Notably, beta diversity analysis revealed that gut-dwelling microbial consortia from *Themis* KO mice clustered separately from those of *Themis* WT mice, indicating distinct overall community compositions associated with each genotype (Fig. 5B). The *Themis* WT group was characterised by a more balanced microbiome on the phylum level, with almost equally abundant *Bacteroidetes* and *Firmicutes*, whereas in *Themis* KO-derived microbiome, the *Firmicutes* phylum dominated gut-dwelling consortia (Fig. 5C). At the genus level, we observed a marked increase in the relative abundance of bacteria belonging to *Clostridium* in the microbiome derived from *Themis* KO mice (Fig. 5D). In contrast, members of the *Parabacteroides* genus exhibited a significantly reduced abundance in the *Themis* KO microbiome compared to WT controls (Fig. 5D). This reciprocal trend suggests a shift in microbial community structure associated with *Themis* deficiency, which may have downstream effects on host metabolism and immune regulation.

### Themis KO-associated microbiota is insufficient by itself to drive T2D

A functional aberration of T cells associated with *Themis* deficiency alters the composition of the microbiome, clearly highlighting the role of T cells in shaping and regulating the intestinal microbiome through the T cell-microbiome interaction axis. At the same time, the reciprocal nature of this interactome suggests that microbiome changes driven by the adaptive immune system can, through positive or negative feedback loops, influence T cell function. This bidirectional crosstalk may further promote pro-inflammatory shifts within the adaptive immune compartment and, consequently, the acceleration of metabolic syndromes in the *Themis* KO model. Thus, to investigate the potential influence of the reshaped microbiome in *Themis* KO mice on the development of T2D, we conventionalized germ-free *Themis*-sufficient B6 mice by orally gavaging them with stool samples collected from either *Themis* KO or *Themis* WT donors. Stool samples were collected after 12–13 weeks of HFD feeding. After two weeks post conventionalization by fecal microbiome transplant (FMT), where animals were kept on chow diet, the recipient mice were placed on HFD (Fig. 5E). To our surprise, germ-free mice colonized with the *Themis* KO microbiome gained much less weight than those receiving the *Themis* WT microbiome, with the exception of the very earliest timepoint (Fig. 5F). Glucose tolerance tests revealed faster glucose clearance in mice colonized with the *Themis* KO microbiome (Fig. 5G), which was contrary to our initial expectations. Despite lower overall body weight at the study endpoint, mice colonized with the *Themis* KO microbiome displayed pronounced metabolic abnormalities. These included significantly increased VAT, enlarged stomach, pancreas, and large intestine, and a reduced liver size compared to mice colonized with the *Themis* WT microbiome (Suppl. Table 1). Blood biochemistry analysis showed decreased levels of alanine transaminase (ALT), total protein (TP), and globulin (GLOB), alongside elevated blood urea nitrogen (BUN) levels in the *Themis* KO FMT group, suggesting possible hepatic and renal inflammation (Suppl. Table 1). We also evaluated T cell infiltration in VAT. While the overall proportions of CD4^+^ and CD8^+^ T cells were comparable between the groups (Suppl. Figure 4), quantification of T cell numbers per gram of fat revealed a modest increase in total, CD4^+^, and CD8^+^ T cell counts in mice colonized with the WT microbiome.

### Themis deficiency-induced changes in the microbiome have long-lasting, irreversible effects

**Figure 6:**
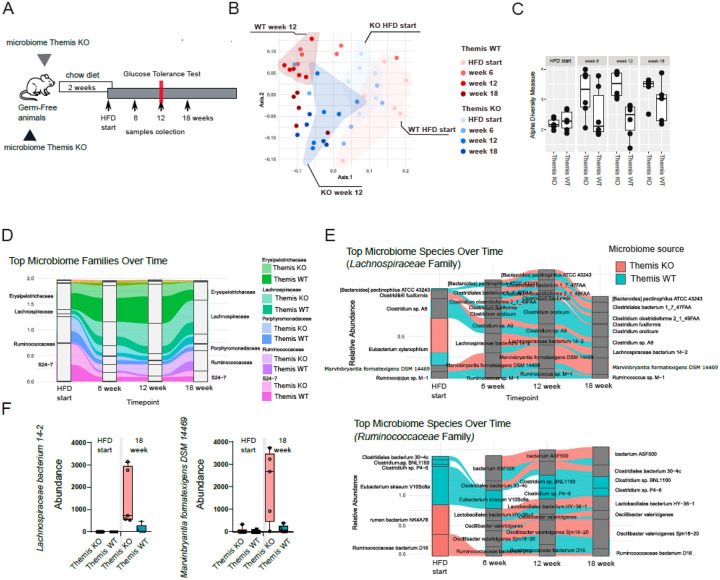
Longitudinal analysis of gut microbiota derived from *Themis* KO and WT mice during HFD exposure **A**. Schematic of experimental design: germ-free animals were colonized with microbiota derived from *Themis* WT and KO, maintained on chow diet, and switched to HFD after 2 weeks. Fecal samples were collected at baseline (HFD start), week 6, 12, and 18. Glucose tolerance tests were performed at week 12. **B**. Beta diversity analysis across timepoints in *Themis* WT and KO groups. **C**. Alpha diversity measures across timepoints in *Themis* WT and KO groups. **D**. Relative abundance of major microbiome families over time. **E**. Abundance across the experiment’s timepoints of representative species within the Lachnospiraceae family (upper) and within the Ruminococcaceae family (lower), **F**. Abundance of *Marvinbryantia formatexigens DSM 14469* and *Lachnospiraceae bacterium 14–2* at the baseline (HFD start) and after 12 weeks of HFD within microbiomes derived from *Themis*WT and KO models.

Collectively, these findings suggest that the microbiome shaped in *Themis* KO animals lacks the capacity to induce T2D when introduced into a *Themis*-sufficient adaptive immune environment in WT B6 hosts. This observation further underscores the central role of T cells themselves as key orchestrators of the inflammatory processes occurring within adipose tissue. At the same time, the observed changes suggesting possible hepatic and renal inflammation in hosts colonized with the *Themis* KO-derived microbiome led us to investigate the temporal dynamics of microbiome composition in these two experimental groups. We collected stool samples for microbiome analysis at baseline, defined as the day the mice were two weeks post-colonization and the diet was switched from chow to HFD, as well as at 6, 12, and 18 weeks following HFD initiation (Fig. 6A). DNA was isolated from stool samples and full-length 16S rRNA gene libraries (covering the V1-V9 regions) were generated. This comprehensive sequencing approach enabled species-level resolution of the bacterial community composition in the gut microbiota. Notably, the microbiomes of WT and *Themis* KO recipients showed substantial differences in taxonomic composition at the baseline time-point and maintained distinct profiles throughout the course of the experiment (Fig. 6B,C). Beta diversity analysis revealed that at the start of HFD feeding, microbial communities derived from *Themis* KO and WT donors clustered separately, indicating a persistent genotype-dependent imprint on microbiome structure following colonization (Fig. 6B).

These differences were even more emphasized at the 12 week timepoint (the day of glucose tolerance testing: Fig. 6A). Interestingly, alpha diversity analysis revealed a similar species richness in *Themis* KO and WT-derived microbiomes at the start of the HFD. However, in the case of the *Themis* KO-derived microbiome (considering the group as a whole), we observed a substantial and progressive increase in alpha diversity across successive time points (Fig. 6C). A similar trend was also present in the group colonized with the *Themis* WT-derived microbiome, though it was less pronounced. These findings suggest that the microbial communities shaped in *Themis* KO mice retain a stable and distinct ecological identity, even after transfer into a *Themis*-sufficient environment. Thus, the functional characteristics of the *Themis* KO-derived microbiome cannot be fully reprogrammed to resemble those of the *Themis* WT-microbiome interaction, highlighting the long-lasting impact of initial immune-driven microbial shaping.

Notably, as previously reported, HFD treatment can increase gut microbiome diversity; however, this effect has not been consistently observed across different studies^22^. Therefore, it can be concluded that the extent to which HFD influences microbiome complexity may depend on the preexisting microbial composition (as in this case), the genetic background of the host or both. Interestingly, in both groups of mice, we observed a dramatic shift in taxonomic composition following the dietary switch from chow to HFD. At baseline, both KO and WT-derived microbiomes exhibited a balanced ratio of *Firmicutes* to *Bacteroidetes*. However, upon HFD initiation, this balance was profoundly disrupted, with both microbial consortia becoming overwhelmingly dominated by *Firmicutes* (Suppl. Figure 5A). At finer taxonomic resolution, we found that the SR24–7 family, previously the dominant representative of the *Bacteroidetes* phylum, underwent the most significant decline after the diet switch (Fig. 6D). In contrast, two families within the *Firmicutes* phylum, *Lachnospiraceae* and *Ruminococcaceae*, significantly increased in abundance in response to the HFD. Notably, the expansion of *Lachnospiraceae* was particularly pronounced in mice colonized with the *Themis* KO-derived microbiome. Importantly, similar changes, such as an altered *Firmicutes*-to-*Bacteroidetes* ratio and an increase in *Lachnospiraceae* and *Ruminococcaceae*, have been consistently reported in mice treated with HFD and in human cohorts with obesity and T2D^22,51,52^.

Despite common trends observed at the family and phylum levels across both animal groups, species-level analysis revealed distinct differences associated with the original host of the transferred microbiome. Within the *Lachnospiraceae* family, *Clostridium* sp. A9 and *Ruminococcus* M1 were significantly more abundant in *Themis* WT-derived microbiomes at baseline, with *Ruminococcus* M1 being undetectable in the *Themis* KO group (Fig. 6E). Interestingly, both species marked their presence more substantially within *Themis* KO-derived microbiomes when animals were switched to HFD. However, this overall trend, more marked in WT than in *Themis* KO, prevailed. The opposite trend was observed in the context of *Marvinbryantia formatexigens* DSM 14469 and *Lachnospiraceae bacterium* 14 – 2 (Fig. 6E, F). Notably *M. formatexigens* produces elaidate, both *in vivo* and *in vitro*. This is a trans-unsaturated fatty acid reported to be involved in the pathology of T2D^21,52^. Interestingly, *Eubacterium xylanophilum*, a dominant bacterium in *Themis* KO-derived microbiomes, completely disappeared from the bacterial communities in both groups following the dietary change. This species is primarily responsible for fermenting complex carbohydrates abundant in the standard chow diet^53^. Therefore, its loss can be attributed to competitive disadvantage resulting from restricted access to its main food source under HFD conditions. A more distinct species composition between the experimental groups was observed within the *Ruminococcaceae* family (Fig. 6E). At baseline, the core of the *Ruminococcaceae* community in WT-derived microbiomes consisted of an almost entirely non-overlapping set of species compared to that in *Themis* KO-derived microbiomes. *Clostridium* sp. P4–6 and *Clostridium* sp. BNL 1100 formed a unique signature in *Themis* KO-derived microbiomes, being present from the initial stages of colonization on the chow diet and progressively increasing in dominance after HFD treatment. Notably, these species were completely absent from *Themis* WT-derived microbiomes throughout the entire duration of the experiment. Some bacterial species that were initially restricted to one genotype of microbiome donors appeared in the opposite experimental group following the HFD. For example, *Ruminococcus bacterium* D16, which was specific to the *Themis* KO group at baseline, emerged in *Themis* WT-derived microbiomes after HFD. Similarly, *Clostridiales bacterium* 30–4C, initially found only in the *Themis* WT group, became common to both groups after the dietary shift. Interestingly *Clostridium citroniae* and *C. bolteae*, previously associated with T2D risk were only present in *Themis* KO-derived microbiome after 12 weeks of HFD (Suppl. Figure 5B).

Our data clearly indicate that *Themis* deficiency has a profound impact on gut microbiome composition through the microbiome-adaptive immune system axis. This was particularly evident during the adaptation of the KO or WT-derived bacterial consortia to a *Themis*-sufficient environment, and subsequently under HFD conditions. Notably, the microbiome shaped in a *Themis* KO host exerts a different effect when transferred to a *Themis* WT host, with no evidence of accelerating the development of T2D. These findings suggest that the kinetics of T2D development depend on the coexistence of permissive factors within both the adaptive immune system and the microbiome.

## Discussion

This study reveals a striking link between *Themis* deficiency and the development of insulin resistance and T2D, uncovering a complex interplay between adaptive immunity and metabolic regulation. While various compartments of the adaptive immune system have been studied in relation to T2D pathogenesis, the Treg subset is increasingly recognized for its dominant immunoregulatory role in maintaining adipose tissue homeostasis. Notably, both quantitative and qualitative aberrations within the Treg compartment have been observed in the VAT of obese mouse models^9 10^. Furthermore, leptin, a dominant adipokine elevated in obesity, has emerged as a key molecular regulator that negatively influences Treg homeostasis and suppresses their proliferation and function in adipose tissues, thereby linking metabolic cues with impaired immune regulation and contributing to the inflammatory milieu that promotes insulin resistance^8,9,54^. Data derived from human studies present a more complex picture. Peripheral blood analyses in obese T2D patients reflect a trend similar to that seen in animal models^7^. However, examination of omental tissue from obese individuals reveals no significant differences in Treg population size compared to non-obese counterparts^6,55^. Crucially, our analysis of Tregs in the context of *Themis* deficiency found no significant variation in Treg numbers or distribution in VAT between *Themis* KO and WT mice. These results suggest that the metabolic dysregulation observed in *Themis* KO models may primarily stem from disruptions in other immune cell subsets rather than changes within the Treg compartment.

CD8^+^ T cells, activated within the adipose tissue microenvironment, have been strongly implicated in initiating obesity-associated inflammation^10^. In HFD induced T2D model, a dominant fraction of VAT-infiltrating CD8^+^ T cells have high expression of CXCR3 and KLRG1^11^. These HFD-induced effector T cells promote the development of low-grade chronic inflammation in adipose tissue by enhanced recruitment and polarisation of M1-like macrophages. Additionally, another proposed mechanism highlights the role of IFNγ-producing CD8^+^ T cells in suppressing beige adipogenesis and so contributing to energy dissipation by modulating catecholaminergic signaling pathways within adipose tissue^14^. Clinical data indicate that, in addition to the expansion of Th1 and Th17 cells, the VAT of obese individuals with T2D harbors an increased population of IFNγ-producing CD8^+^ T cells^6^.

We have demonstrated that *Themis* KO mice exhibit an accelerated onset of insulin resistance when fed HFD, despite the lack of quantitative differences regarding infiltration of pathogenic IFNγ-producing CD8^+^ T cells in VAT compared with its WT counterpart. The phenotype in *Themis*-deficient mice seems to arise, not from increased inflammatory T cell presence, but rather from a functionally altered T cell population, pointing to a dysregulated immune environment. A peripheral knockout model further confirmed that the observed metabolic dysfunction stems from extrathymic T cell alterations rather than defects during thymic selection. Interestingly, antibody-mediated depletion of T cells, CD3^+^ or more specifically CD8^+^ T subsets, reversed insulin resistance, underscoring the central role of CD8^+^ T cells in the disease’s etiology observed in the *Themis* KO model. The direct mechanism of pathogenic T cell recruitment or polarisation within adipose tissue is still unknown. However, it has been proposed that the recognition of self-antigens may serve as a potential trigger for the development of metabolic syndrome, aligning with the broader hypothesis that a subset of T2D patients may exhibit autoimmune features^2,4,56^. Supporting this notion, associations have been identified between T2D-related metabolic traits and specific HLA class II alleles encoding MHC-II molecules involved in antigen presentation to T cells. Specifically, the absence of the DRB5 allele has been linked to increased T2D risk, whereas alleles such as HLA-DQA01, HLA-DQB06, and HLA-DRB1 appear to exert a protective effect^57^. Importantly, TCR repertoire remodeling has been reported during the onset of T2D, marked by distinctive alterations within the CDR3 region, crucial for antigen recognition, along with biased usage of specific Vβ (TRBV7–8) segments among T cells in affected individuals^58^.

We have shown the unique reshaping of the CD8^+^ TCR repertoire within adipose tissue, particularly in *Themis* KO mice. The site-specific expansion of clonotypes expressing TRAV6D-6 and TRAV12–2 segments points to an *in situ* antigen-driven activation process. Biophysical analysis of CDR3 regions revealed distinct patterns of hydrophilicity, length, and polarity, suggesting selection within the VAT for TCRs optimized for recognition of adipose tissue-associated antigens. Importantly, physical features-based expansion of TCR clones was emphasised within the VAT of *Themis* KO. The expansion of TCR clones in *Themis* KO mice was driven by distinctive biophysical features and was strongly emphasized within VAT. Compared to WT counterparts, *Themis* KO mice exhibited a markedly higher number of expanded clonotypes, indicating a broader responsive TCR repertoire. We believe this phenotype reflects a lowered activation threshold in *Themis*-deficient T cells, which allows for activation and expansion of multiple clones with similar TCR-MHC affinity. In turn, this relaxed TCR-driven activation in *Themis* KO animals likely permits broader recognition of adipose tissue-associated antigens, fostering a state of hyper-responsiveness and clonal dominance. This heightened immunological activity may contribute to the faster kinetics observed in the progression of T2D within *Themis* KO mice, suggesting an immunologically driven mechanism accelerating metabolic dysfunction. Conversely, TRAV9N-3/Vα3.2 TCRs, known for their non-classical MHC recognition^49^, did not show signs of clonal expansion or antigen-driven reshaping in either genotype. This reinforces the notion that the dominant immune response in VAT during early T2D pathogenesis is restricted to classical MHC class I-restricted CD8^+^ T cells, likely activated by adipose tissue-derived protein antigen.

The influence of host-resident microbial consortia on the immune system, particularly the adaptive immune system, has been extensively investigated^15,16,59,60^. These studies reveal a diverse array of mechanisms employed by distinct members of the microbiome community to modulate host responses and support immune homeostasis. The *in situ* progression of inflammation is thought to be a primary driver of insulin resistance during the onset of T2D. Concurrently, hallmarks within immune compartments associated with obesity-induced inflammation are accompanied by profound alterations in the gut-resident microbiome, which resides anatomically distant from the VAT compartment^22,51,61^. An important question remains unsolved: whether ecological changes within the microbiome primarily represent a response to dietary factors, and how such shifts in microbial communities contribute to the proinflammatory response within VAT during the development of metabolic syndrome. Our study highlights the profound impact of *Themis* deficiency on the gut microbiota. *Themis* KO mice developed a distinct microbial composition characterized by reduced alpha diversity and increased abundance of *Firmicutes*, particularly *Clostridium* species. These findings suggest that, at least under steady-state conditions, the directionality of microbiome-immune system interaction predominantly favors immune system-driven modulation of the microbiome. Notably, T cells appear to exert a more substantial influence on shaping microbial communities than does the microbiome on T cell-mediated immune responses. This notion was further supported when *Themis* KO-derived microbiome was transferred into germ-free WT mice, but failed to recapitulate the full metabolic phenotype. We have shown that both *Themis* WT and *Themis*-KO-derived microbiomes undergo remodeling upon a shift from standard chow to HFD. The diet-induced restructuring of gut-resident microbial consortia in our experimental groups closely mirrored trends previously reported in HFD-treated mouse models and obese human individuals^20,22,52,53,61^. The relative decrease in abundance of *Bacteroidetes* constitutes a hallmark of obesity in both human and animal models. Studies involving human cohorts have identified several bacterial species as risk factors for the development of incipient T2D^19,62,63^. According to these studies *Clostridium citroniae* and *C. bolteae* are recognized as microbial signatures associated with elevated risk for T2D^19,62^. In our model, both species were exclusively detected within the HFD-affected *Themis* KO microbiome. Their presence did not influence the kinetics of T2D development observed in our model. Instead, the appearance of both bacterial species was associated with an alleviation of metabolic syndrome features. These findings suggest that, although previously implicated in T2D risk, they may not exert a dominant pathogenic role within the context of our experimental system. We propose that alterations in the microbiome may reflect a functional adaptation to dietary or environmental pressures^20^ rather than direct pathogenic effects. This insight highlights the importance of host genetic context in shaping disease outcomes beyond microbial presence alone. Based on our data we suggest that microbial dysbiosis alone is insufficient to drive T2D development in the absence of *Themis*-deficient T cells. Instead, this points to a synergistic requirement for both immune dysfunction and microbial alterations. These findings underscore the need for a more holistic approach to understanding T2D pathogenesis, one that integrates dietary, microbial, and host genetic factors. The *Themis*-deficient model reveals that, in certain cases, intrinsic immune dysfunction, rooted in germline-encoded defects, may play a central role in the onset and progression of T2D. Therefore, further investigations into the immune landscape, including targeted screening for germline abnormalities, are essential to uncover previously overlooked mechanisms contributing to metabolic disease.

## Materials and Methods

### Mice

Themis^−/−^Foxp3-GFP, Themis^+/+^Foxp3-GFP, Themis^*fl*/*fl*^.d/Lck-cre^+^, Themis^*fl*/*fl*^.d/Lck-cre^−^, all on C57BL/6 background were bred in restricted flora (RF) facilities at Comparative Medicine, NUS. All animal procedures were approved by NUS IACUC.

### Dietary interventions in mice

At 6 weeks of age, mice from either genotype were randomly grouped into cages of 3–5 mice each and were fed either HFD or ND (11–12 mice per group). The mice were fed twice a week and cages changed once a week. The weight of the mice was noted weekly. High fat diet (HFD) animals were fed a diet of 45 kcal% fat (Research Diets Inc, New Jersey, USA). Normal chow diet (ND) animals were fed a diet containing 6 kcal% fat (Harlan Teklad laboratory animal Diets, Envigo, New Jersey, USA).

### Conventionalization of germ-free mice

All germ-free experiments were performed at the SingHealth Experimental Medicine Centre SEMC, Singapore General Hospital SGH. Germ-free mice (C57BL/6 background) were randomly grouped into two groups of five for conventionalization. 3–4 stool pellets were collected per genotype from *Themis* WT and *Themis* KO mice at 12–13 weeks of HFD and transported in anaerobic bags within an hour from NUS CM to SGH SEMC. The pellets were then dissolved in 3 ml sterile PBS (Hyclone, Utah, USA). 500 μl of the stool suspension was aliquoted and stored at −80°C for later microbiome analysis. 150 μl of the stool suspension was administered to each germ-free mouse via oral gavage. The remaining stool suspension was sprayed onto the germ-free mice that were conventionalized with the microbiome of the respective genotype. This process of conventionalization was repeated twice a week for two weeks. After conventionalization, mice were put on HFD for the next 34 weeks.

### T cell depletion experiments

To assess the effects of depletion of T cells or CD8^+^ T cells on preestablished adipose inflammation in diet induced obesity mice, we fed *Themis* KO mice on HFD for 16 weeks. After that, mice were injected with the respective antibodies. For total T cell depletion, we injected 150 μg of anti-CD3e F(ab’)_2_ (Bio X Cell, New Hampshire, USA) or isotype control in 150 μl of PBS intraperitoneally for 5 consecutive days. For CD8^+^ T cell depletion, we intraperitoneally administered either CD8-specific antibody (120 μg per mouse; Biolegend, California, USA) or control IgG three times per week for 2 weeks (total of six administrations). At 24 weeks of HFD, we performed oral glucose and insulin tolerance tests and then euthanised the mice for analysis of their adipose tissue.

### Histology

The tissue samples were stored in 5ml 4% Para-Formaldehyde (PFA; Sigma-Aldrich, Missouri, USA) at 4°C for 1–7 days. The samples were then transferred into tissue cassettes (Thomas Scientific Inc., New Jersey, USA) and stored in 70% ethanol (Sigma-Aldrich, Missouri, USA) at 4°C until further processing. For processing the tissue samples for sectioning, the samples were put in an automated tissue processor (Leica Biosystems, Wetzlar, Germany) with the following program: 80% ethanol for 1 hour, 95% ethanol for 1 hour, 3 times 100% ethanol for 1.5 hours each, 3 times xylene (Sigma-Aldrich, Missouri, USA) for 2.5 hours each, 1:1 (paraffin: xylene) for 2.5 hours, paraffin for 2.5 hours then paraffin (Sigma-Aldrich, Missouri, USA) until further use. After this processing, the tissue samples were embedded into paraffin using Histocore Arcadia H (Leica Biosystems, Wetzlar, Germany). These embedded samples were then cooled overnight on Histocore Arcadia C (Leica Biosystems, Wetzlar, Germany). The embedded samples were then cut into 5 μm sections using a Leica RM 2255 microtome (Leica Biosystems, Wetzlar, Germany) and transferred onto l-lysine slides (ThermoFisher Scientific, Massachusetts, USA). The slides were then rested overnight at room temperature before storage. Hematoxylin and Eosin staining was performed for all the sections using the following procedure. The slides were dewaxed in xylene for 10 minutes and then rehydrated in ethanol: twice in 100% ethanol for 2 minutes, 95% ethanol for 2 minutes and 70% ethanol for 2 minutes. The slides were then stained in Harris Hematoxylin (Leica Biosystems, Wetzlar, Germany) for 5–7 minutes. Then washed in distilled water 3 times. The slides were then left to blue in tap water for 2 minutes, followed by a wash in distilled water. They were then dehydrated in 70% ethanol for 1 minute, followed by staining in Eosin (Sigma-Aldrich, Missouri, USA) for 30 seconds (10 dips for VAT sections and 3 dips for liver sections). They were then washed in 95% ethanol for 1 minute, followed by two washes in 100% ethanol for 1 minute each, followed by xylene for 10 minutes. The slides were then left to dry overnight. The sections were covered with 1–2 drops of Histomount (ThermoFisher Scientific, Massachusetts, USA) the next day and covered with coverslips and again dried overnight. The sections were then viewed under a Leica DM 2000 light microscope (Leica Biosystems, Wetzlar, Germany) and images were taken. Images were analysed using ImageJ software for cell size and cell numbers.

### Glucose Tolerance

Glucose Tolerance Test (GTT) was performed on these mice at 8,10 and 12 weeks of age. For GTTs, glucose (1–2 mg/g body weight; Sigma-Aldrich, Missouri, USA) was administered through intraperitoneal (i.p.) injection after fasting the mice overnight for 16 hours. Blood glucose levels were measured before, 15, 30, 45, 60, 75, 90, 105, and 120 minutes after injection. The tails of the mice were snipped and gently massaged to produce the blood drop, which was then analysed by a glucometer (Roche Diagnostics One Touch, Risch-Rotkreuz, Switzerland) to produce the blood glucose readings.

### Insulin Tolerance

Insulin Tolerance Test (ITT) was performed on these mice at 24 weeks of age. For ITTs, Insulin (0.75IU Insulin/g body weight) (Sigma-Aldrich, Missouri, USA) was administered through intraperitoneal (i.p.) injection after fasting the mice overnight for 6 hours. Blood glucose levels were measured before, 15, 30, 45, 60, 75, 90, 105, and 120 minutes after injection. The tails of the mice were snipped and gently massaged to produce the blood drop, which was then analysed by a glucometer to produce the blood glucose readings.

### Isolation of Stromal Vascular Fraction (SVF)

VAT was cut into smaller pieces in 3ml FWB and digested for 20 minutes with 3 mL Collagenase II (4mg/mL) (Sigma-Aldrich, Missouri, USA) containing 10mM CaCl_2_ (SigmaAldrich, Missouri, USA) at 37°C on an orbital shaker. 10 ml of FWB was added to this mixture and titurated multiple times with a pipette to get a homogenous suspension. The suspension was passed through a 70μm sieve to remove any clumps and centrifuged at 300g for 10 minutes at 4°C. The resulting cell pellet was resuspended in 3 ml ACK lysis buffer for 10 minutes to lyse the RBCs. RBC lysis was stopped by adding 12 ml VFWB to each sample and the samples were then centrifuged at 300g for 10 minutes at 4°C. The obtained stromal vascular fraction was resuspended in 1ml VFWB.

### Intracellular cytokine analysis

0.5 ml of the SVF cell suspension and splenocytes was used for stimulation with phorbol myristate acetate (PMA; 50 ng/ml; Sigma-Aldrich, Missouri, USA) and ionomycin (500 ng/ml; SigmaAldrich, Missouri, USA) for 4–6 hrs at 37°C and adding Golgistop (Brefeldin A) (BD Biosciences, California, USA) in a 6 well plate. Cells were then transferred to 5ml FACS tubes and pelleted by centrifugation at 500g for 5 minutes to remove the media. Cells were then stained with mAbs specific for CD4, CD8, TCRb and CD25 for 30 minutes on ice, followed by a wash with VFWB. The supernatant was discarded, and the cells were then resuspended in 0.2 ml IC fixation buffer (eBiosciences, California, USA) while being vortexed, followed by incubation at room temperature for 20 minutes. The cells were then washed twice with 2 ml 1X permeabilization buffer (eBiosciences, California, USA), followed by intracellular staining for TNF, IFNγ, and IL2 at room temperature for 30 minutes. The cells were then washed once with 2ml 1X permeabilization buffer and then with 2ml VFWB. The cells were then resuspended in 300 μl FWB for analysis on a flow cytometer. 25 μl Count Bright beads were added to each sample for cell count analysis.

### Flow cytometry

For surface staining, cell pellets were resuspended in 100 μl FWB, containing the fluorophore-conjugated antibodies and incubated on ice for 30 minutes in the dark. Cells were then centrifuged at 1200 rpm at 4°C for 5 minutes and resuspended in 300 μl of FWB for flow cytometry analysis. Cells were analysed on BD LSR Fortessa X-20 flow cytometer (BD Biosciences, California, USA). Flow cytometry data was analyzed using FlowJo software (Treestar, California, USA).

### RNA isolation

Tissue samples 0.4–0.5g of VAT were cut into very small pieces and added to ceramic beads (Omni Inc., Georgia, USA) in an omni tube (Omni Inc., Georgia, USA). 1ml Trizol (Sigma-Aldrich, Missouri, USA) was added to the VAT. The samples were then homogenized in an Omni Bead ruptor 24 (Omni Inc., Georgia, USA) kept in the cold room using the following program: speed 5.3m/s for 45 sec followed by 1 minute rest on ice followed by another cycle of 5.3m/s for 45seconds. The homogenized lysate was then transferred to a fresh Eppendorf tube. 200μl chloroform (Sigma-Aldrich, Missouri, USA) was added to separate the aqueous and organic phase. The sample was mixed vigorously and incubated at room temperature for 3 minutes. The sample was then spun at 12000g for 15 minutes at 4°C. The aqueous layer was then separated, and an equal volume of 70% ethanol was added to precipitate the total RNA. RNA isolation kit (MACHAREY-NAGEL, Germany) was then used to isolate RNA as follows: The sample was mixed gently and up to 750μl of the mixture was transferred to the RNA column which was then spun at 11000g for 30 seconds. This process was repeated until all of the mixture was passed through the column. The column was then washed with 350μl MDB buffer for 1 minute at 11000g. To remove the contaminating DNA, 95μl of freshly prepared rDNAse (10μl rDNAse + 90μl reaction mix) mixture was added to the column and incubated at room temperature for 15 minutes. To stop the reaction, 200μl RA2 was added to the column and centrifuged for 30 seconds at 11000g. The column was then placed into a fresh tube, 600μl RA3 was added to wash the samples and centrifuged at 11000g for 30 seconds. The column was then placed into a fresh tube, 250μl RA3 was added to wash the samples and centrifuged at 11000g for 30 seconds. The flow through was discarded and the column was spun for 2 minutes at 11000g to remove all the residual ethanol. To elute the RNA, 40μl nuclease free water was added to the column and incubated for 5 minutes and then centrifuged at 12000g for 2 minutes at 4°C. The RNA samples were then quantified using a ND1000 (ThermoFisher Scientific, Massachusetts, USA) and stored at −80°C until further analysis.

### Sequencing of TCRα repertoires

To generate NGS libraries encompassing the full TCR repertoire, total RNA was extracted from equivalent numbers of sorted single-positive CD8^+^ thymocytes, lymph node-derived CD8^+^ T cells, and CD8^+^ T cells residing in adipose tissue. Reverse transcription was carried out using a previously established protocol, incorporating template-switching primers (TAAGAGACAGCAACTACTACTGCrGrGrG, with ‘r’ denoting ribonucleotides). The resulting cDNA underwent two rounds of amplification using Q5^®^ High-Fidelity DNA Polymerase (New England Biolabs, MA, USA), following the manufacturer’s guidelines. The first PCR utilized primers tcgtcggcagcgtcagatgtgtataagagacagcaactactACTGC and GTCTCGTGGGCTCGGAGATGTGTATAAGAGACAGggtacacagcaggttctgg. The second round employed indexed primers CAAGCAGAAGACGGCATACGAGAT[i7]GTCTCGTGGGCTCGG and AATGATACGGCGACCACCGAGATCTACAC[i5]TCGTCGGCAGCGTC, where i7 and i5 correspond to Illumina Nextera V2 index sequences (Illumina, CA, USA). Library purification was performed using AMPure XP beads (Beckman Coulter, CA, USA), and amplicon concentrations were measured with both the Qubit DNA Assay (Thermo Fisher Scientific, MA, USA) and the KAPA Library Quantification Kit (Kapa Biosystems, MA, USA). Sequencing was conducted on the MiSeq platform using MiSeq Reagent Kits v2 (Illumina, CA, USA). Extraction of the sequences corresponding to the TCRs was performed using MiXCR platform^64^. Further processing of data was done using VDJTools software^65^.

### Whole 16S rDNA sequencing

To analyse remodelling process of distribution of the bacterial species over the timespan of the experiment, total DNA was isolated from mouse stool pellets collected in four timepoints; start of HFD, 6, 12, and 18 weeks after HFD introduction. Pellets were used directly for genomic DNA isolation using Microbiome DNA Purification Kit. Using the DNA template, the PCR was carried out using Q5 high fidelity polymerase with primers for complete 16S rDNA amplification:

### V1: TCGTCGGCAGCGTCAGATGTGTATAAGAGACAGAGRGTTTGA

TYMTGGCTCAG.

### V9: GTCTCGTGGGCTCGGAGATGTGTATAAGAGACAGGGYTACCTTGTTACGACTT

The obtained amplicons were purified using AMPure XP beads. DNA concentration was quantified using Qubit DNA quantification assays (Invitrogen). The tagmentation, library barcoding, and amplification were carried out using Nextera XT DNA Library Preparation Kit (Illumina), and libraries were sequenced on MiSeq instrument using MiSeq Reagent Kit v3 (600-cycle) (Illumina). The raw reads were *de novo* assembled using MATAM^66^ and aligned to the SSU database (SILVA 138.1 release). Reconstructed SSU were then annotated to the individual bacteria species using METAXA2^67^.

### Statistical analysis

Statistical analyses were performed using R (version R4.5.1), GraphPad Prism (version 9.5), and Microsoft Excel, selected based on the specific requirements of each dataset and analytical task. Data were routinely presented as means ± standard deviation (s.d.), and we determined significance by Student’s t test or Mann-Whitney U test (as indicated). We considered a P value of equal to or less than 0.05 as statistically significant.

## Supplementary Material

Supplementary Files

This is a list of supplementary files associated with this preprint. Click to download.

- Suppfigandtable.pdf
